# Multi-kingdom metagenomic characterization of the gut bacteriome, mycobiome, and virome in chronic functional constipation

**DOI:** 10.3389/fcimb.2026.1744020

**Published:** 2026-03-23

**Authors:** Wen Sun, Yue Li, Jinwen Su, Shanliang Mao, Shuang Yang, Yindi Zhu, Yunhua Liu, Jie Ma, Wei You, Yan Zhang, Hongxing Guo, Guorui Xing, Shenghui Li, Qiulong Yan, Xingyi Ma

**Affiliations:** 1 Centre for Translational Medicine, Shenzhen Bao’an Chinese Medicine Hospital, Guangzhou University of Chinese Medicine, Shenzhen, China; 2 Puensum Genetech Institute, Wuhan, China; 3 Department of Pediatrics, Loudi Health Center for Women and Children, Loudi, China; 4 Department of Traditional Chinese Medicine, Suining Central Hospital, Suining, China; 5 School of Chinese Medicine, Wenzhou Medical University, Wenzhou, China; 6 International Acupuncture and Moxibustion Innovation Institute, School of Acupuncture-Moxibustion and Tuina, Beijing University of Chinese Medicine, Beijing, China; 7 School of Traditional Chinese Medicine, Beijing University of Chinese Medicine, Beijing, China; 8 Department of Acupuncture and Moxibustion, Beijing Hospital of Traditional Chinese Medicine, Capital Medical University, Beijing, China; 9 Department of Traditional Chinese Medicine, Beijing Friendship Hospital, Capital Medical University, Beijing, China; 10 The Fifth Affiliated Hospital of Southern Medical University, Guangzhou, China; 11 School of Biomedical Engineering & School of Science, Harbin Institute of Technology, Shenzhen, China; 12 Key Laboratory of Science and Engineering for the Multi-modal Prevention and Control of Major Chronic Diseases, Ministry of Industry and Information Technology, Harbin Institute of Technology (HIT) Zhengzhou Research Institute, Zhengzhou, China; 13 Biosen International and Briteley Institute of Life Sciences, Yantai, China

**Keywords:** chronic functional constipation, gut microbiome, metagenomics, microbial dysbiosis, multi-kingdom microbiota

## Abstract

**Background:**

Chronic functional constipation (CFC) is a common gastrointestinal disorder increasingly linked to gut microbiome dysbiosis. However, multi-kingdom metagenomic characterization of bacterial, fungal, and viral communities in CFC remains limited.

**Methods:**

Fecal samples from 53 CFC patients and 48 healthy controls were analyzed using whole-metagenome shotgun sequencing. Microbial composition, function, cross-kingdom interactions, and diagnostic potential were evaluated using diversity analyses, KEGG annotation, network analysis, and random forest modeling.

**Results:**

Compared with healthy controls, CFC patients exhibited marked alterations across multiple microbial kingdoms. The gut bacteriome showed significant community-structure shifts despite comparable α-diversity, characterized by depletion of health-associated Firmicutes (e.g., *Faecalibacterium* and *Roseburia*) and enrichment of Proteobacteria (e.g., *Klebsiella*). The mycobiome displayed selective changes in diversity and composition, with several potentially pathogenic fungal taxa enriched in CFC (e.g., *Fusarium* sp. *c181*). In the virome, community composition differed significantly between groups, with higher viral richness in CFC and widespread depletion of diverse bacteriophages in CFC patients. Functional profiling suggested feature-level functional differences without a clear global shift, including reduced carbohydrate transport and utilization pathways and relatively higher abundance of stress-response and metabolic adaptation modules in CFC. Cross-kingdom network analysis demonstrated substantially denser microbial interactions in CFC, dominated by viral associations, with *Faecalibacterium prausnitzii* and *Faecalibacterium*_SGB15346 acting as central hubs. Machine-learning models showed strong discriminatory power for CFC classification based on bacterial and viral features, whereas fungal features contributed less.

**Conclusions:**

CFC is associated with coordinated multi-kingdom gut microbiome dysbiosis involving bacteria, fungi, and viruses, accompanied by functional shifts and intensified cross-kingdom interactions. Bacterial and viral signatures show strong potential as microbiome-based biomarkers for CFC, highlighting the importance of integrating multi-kingdom analyses to better understand disease-associated gut ecosystem alterations.

## Introduction

Constipation is a common gastrointestinal disorder characterized by infrequent bowel movements, difficulty in stool passage, or a persistent sensation of incomplete evacuation. Acute constipation can progress to intestinal obstruction and, in severe cases, may necessitate surgical intervention (Benninga et al., 2005). Among its subtypes, chronic functional constipation (CFC) is the most prevalent form and is defined by the absence of identifiable organic or structural abnormalities. CFC is primarily associated with lifestyle factors, dietary habits, and altered gut function (Camilleri et al., 2017). Epidemiological studies indicate that CFC affects approximately 10–30% of the global population, with prevalence continuing to rise (Lembo and Camilleri, 2003; Suares and Ford, 2011). In addition to impairing quality of life, CFC imposes a substantial economic burden on healthcare systems worldwide (Pinto Sanchez and Bercik, 2011; Sbahi and Cash, 2015).

The gut microbiota comprises a complex community of microorganisms inhabiting the gastrointestinal tract, including bacteria (bacteriome), fungi (mycobiome), and viruses (virome). Although the bacteriome represents the most abundant and diverse component, accumulating evidence suggests that the mycobiome and virome also play essential roles in maintaining intestinal homeostasis (Turnbaugh et al., 2007; Huse et al., 2012). Collectively, the gut microbiota contributes to host health by facilitating dietary fiber fermentation, producing short-chain fatty acids that nourish intestinal epithelial cells and reinforce gut barrier integrity (Flint et al., 2012). Moreover, it modulates mucosal and systemic immune responses, limits pathogen colonization, and supports immune tolerance (Belkaid and Hand, 2014; Di Tommaso et al., 2021). Disruption of this balanced ecosystem, termed gut dysbiosis, has been increasingly implicated in the pathogenesis of CFC, where patients frequently exhibit reduced levels of beneficial microorganisms alongside the expansion of potentially harmful taxa (Ohkusa et al., 2019; Pan et al., 2022). Beyond constipation, gut dysbiosis has also been linked to other gastrointestinal disorders, including inflammatory bowel disease and irritable bowel syndrome (Nagalingam and Lynch, 2012; Öhman and Simrén, 2013), underscoring its broad clinical relevance.

Metagenomics, which enables culture-independent analysis of the collective genomes of microbial communities, has emerged as a powerful approach for investigating gut microbiota composition and function. By directly sequencing microbial DNA extracted from biological samples, metagenomics allows comprehensive characterization of microbial diversity, metabolic potential, and ecological interactions (Handelsman, 2004; Thomas et al., 2012) (Qin et al., 2010). In recent years, metagenomic studies have revealed pronounced microbial alterations in patients with constipation. Compared with healthy individuals, patients with functional constipation often display reduced abundances of beneficial bacteria such as *Bifidobacterium* and *Lactobacillus*, accompanied by enrichment of methanogenic microorganisms that may contribute to delayed intestinal transit and stool hardening (Zhao and Yu, 2016; Yang et al., 2022). Functional analyses further indicate increased representation of genes involved in hydrogen production, methanogenesis, and glycerol degradation within the gut microbiome of constipated individuals (Mancabelli et al., 2017). These findings highlight the advantage of metagenomics over traditional methods by enabling simultaneous taxonomic and functional profiling of complex microbial ecosystems.

Despite these advances, comprehensive multi-kingdom investigations of the gut microbiota in CFC remain limited. To address this gap, we performed high-throughput shotgun metagenomic sequencing of fecal samples from 53 patients with CFC and 48 healthy controls, systematically characterizing alterations in the gut bacteriome, mycobiome, and virome. In addition, we explored inter-kingdom microbial interactions and their associations with clinical features of CFC. This integrative analysis aims to deepen our understanding of the microbial mechanisms underlying CFC and to provide novel insights into potential microbiota-based strategies for its prevention and treatment.

## Methods

### Subject recruitment

We enrolled 53 patients with chronic functional constipation (CFC) and 48 healthy controls (HC) from the Second Affiliated Hospital of Dalian Medical University. CFC was diagnosed by physicians according to the Rome IV criteria. All participants provided written informed consent prior to fecal sample collection. Participants were excluded if they had: (1) metabolic disorders; (2) alcohol consumption within 14 days before sampling; (3) current medication use; or (4) use of antibiotics or probiotics within 30 days before sampling. Healthy controls were recruited to match the patient cohort in key demographic characteristics. This study received approval from the Ethics Committee of the First Affiliated Hospital of DalianMedical University (YJ-KS-KY-2019-93), and all participants provided written informed consent to participate in the study.

### Sample collection, DNA extraction and whole-metagenome shotgun sequencing

Fecal samples were self-collected by patients after defecation in the hospital and immediately placed on dry ice. The samples were then transported to the laboratory, divided into two portions, and placed into separate cryotubes. All fecal samples were stored at -80°C. Total DNA from fecal samples (170 mg per sample) was extracted using the Tiangen fecal DNA extraction kit (Tiangen, China) following the manufacturer’s instructions. DNA concentration and purity were assessed using a NanoDrop2000 spectrophotometer and Qubit 4.0 fluorometer. The total DNA was then fragmented using a Covaris M220 ultrasonicator (Gene Company Limited, China). A 150-bp paired-end library with an insert size of approximately 350 bp was constructed for each DNA sample. All libraries were barcoded and pooled for whole-metagenome shotgun sequencing on the Illumina NovaSeq platform. Initial base calling of the metagenomic dataset was conducted using the system’s default parameters on the sequencing platform. The raw sequencing reads for each sample were processed independently for quality control with fastp (Chen et al., 2018). Fastp trimmed low-quality bases (Q < 30) from the ends of the reads and filtered out reads containing N bases, adapter contamination, or reads shorter than 90 bp, yielding high-quality reads. Human reads were removed from the high-quality reads using Bowtie2 (Langmead and Salzberg, 2012) alignment to the human reference genome (GRCh38).

### Gut bacteriome profiling

The prokaryotic composition of the gut microbiome in fecal metagenomes from all samples was profiled using the MetaPhlAn4 algorithm (Blanco-Míguez et al., 2023). The relative abundances of microbial species were determined by normalizing each sample. Subsequently, the relative abundances at the phylum and genus levels were obtained by aggregating the species abundances within each respective taxon.

### Reference database construction and gut mycobiome profiling

In June 2024, the available fungal genomes from the NCBI database were downloaded, initially comprising 16,634 genomes. Out of these, 1,384 genomes were excluded due to either extremely low assembly quality (N50 length < 2,000 bp or number of scaffolds > 10,000) or the presence of multiple genome mixtures. Consequently, 15,250 high-quality genomes were retained for further analysis (Yan et al., 2024). To reduce the impact of non-specific read mapping to fungal genomes in subsequent analyses, we mapped the filtered reads against three databases, the GRCh38 genome, the UHGG collection, and the SILVA rRNA database (Quast et al., 2012). This step allowed us to exclude reads originating from human or prokaryotic sources. For each sample, the remaining reads were aligned against our customized catalog of gut fungal genomes using Bowtie2 (Langmead and Salzberg, 2012), and the read counts for each genome were computed. To generate mycobiome composition profiles, the read count for each genome was normalized by dividing it by the genome size. The normalized read counts were then summed within each sample to maintain the relative abundance of each fungal population. For different fungal taxa, the relative abundance of a taxon was calculated as the sum of the relative abundances of all populations assigned to that taxon.

### Gut virome profiling

A comprehensive gut virus catalog, termed the Chinese Gut Viral Catalog (cnGVC) (Yan et al., 2025), was constructed from over 10,000 publicly available fecal metagenomes and includes more than 67,000 nonredundant viral operational taxonomic units (vOTUs). High-quality reads from all samples were mapped to the cnGVC database using Bowtie2, with a nucleotide similarity threshold of 95% to define viral “species-level” distinctions (Gregory et al., 2019). To generate the abundance profile of vOTUs in each fecal sample, we aggregated the reads mapped to each vOTU and normalized the relative abundance by the total number of mapped reads in each sample. Subsequently, the relative abundance at the viral family level was determined by aggregating the relative abundances of vOTUs assigned to the same family.

### Gut microbiome functional profiling

#### Microbial functional profiling

Functional profiling of the metagenomes was performed by mapping clean reads to the integrated gene catalog of the human gut microbiome (Almeida et al., 2021), followed by aggregation of counts for orthologous groups (KOs) within the Kyoto Encyclopedia of Genes and Genomes (KEGG) database (Kanehisa et al., 2014). Finally, the abundance of functional modules was determined by summing the read counts across KO members, as defined by KEGG.

### Construction of multi-kingdom co-occurrence network

Bacterial-fungal-vOTU interactions were analyzed using Spearman’s rank correlation with a stringent threshold (|r| > 0.6). Only taxa with a prevalence greater than 10% were included in the correlation analysis. The Benjamini-Hochberg (BH) method was utilized for p-value correction to improve the reliability of the results. Significant associations were visualized as interaction networks through Cytoscape v3.8.2 (Su et al., 2014).

### Statistical analyses

Statistical analyses were performed using R v4.0.1 platform. Principal coordinates analysis (PCoA) based on Bray-Curtis distances was conducted with the *vegan* package (Oksanen et al., 2007). Permutational Multivariate Analysis of Variance (PERMANOVA) was performed using the adonis function in the *vegan* package (Oksanen et al., 2007), with *P*-values calculated from 1,000 permutations. Random forest models were trained using the *randomForest* package (Liaw and Wiener, 2002) with 1,000 trees to classify CFC patients and HC based on the abundance profiles of differential bacteria, fungi, and virus. Model performance was evaluated using receiver operating characteristic (ROC) analysis implemented with the *pROC* package (Robin et al., 2011). The Wilcoxon rank-sum test was implemented using the function *wilcox.test*. Student’s t-test was implemented using the function *t.test*. Linear Discriminant Analysis Effect Size (LEfSe) analysis was performed using the LEfSe Conda v1.1.2 based on classification across all taxonomic levels (Segata et al., 2011). Data visualizations were created using the *ggplot2* package (Wickham et al., 2016).

## Results

### Study population and basic characteristics

This study enrolled 53 patients with CFC and 48 age-matched HC from the Second Affiliated Hospital of Dalian Medical University. As summarized in **Supplementary Table 1**, the two cohorts exhibited comparable demographic characteristics. The mean age did not differ significantly between CFC patients (59 ± 5 years) and HC participants (60 ± 6 years) (Student’s t test, p = 0.264). Body mass index (BMI) was also similar between groups (CFC: 23.1 ± 2.5 kg/m² vs. HC: 23.1 ± 2.8 kg/m², p = 0.934). In addition, no significant difference in gender distribution was observed (p = 0.125).

### Characteristics of the gut bacteriome in patients with CFC

**Figure 1:**
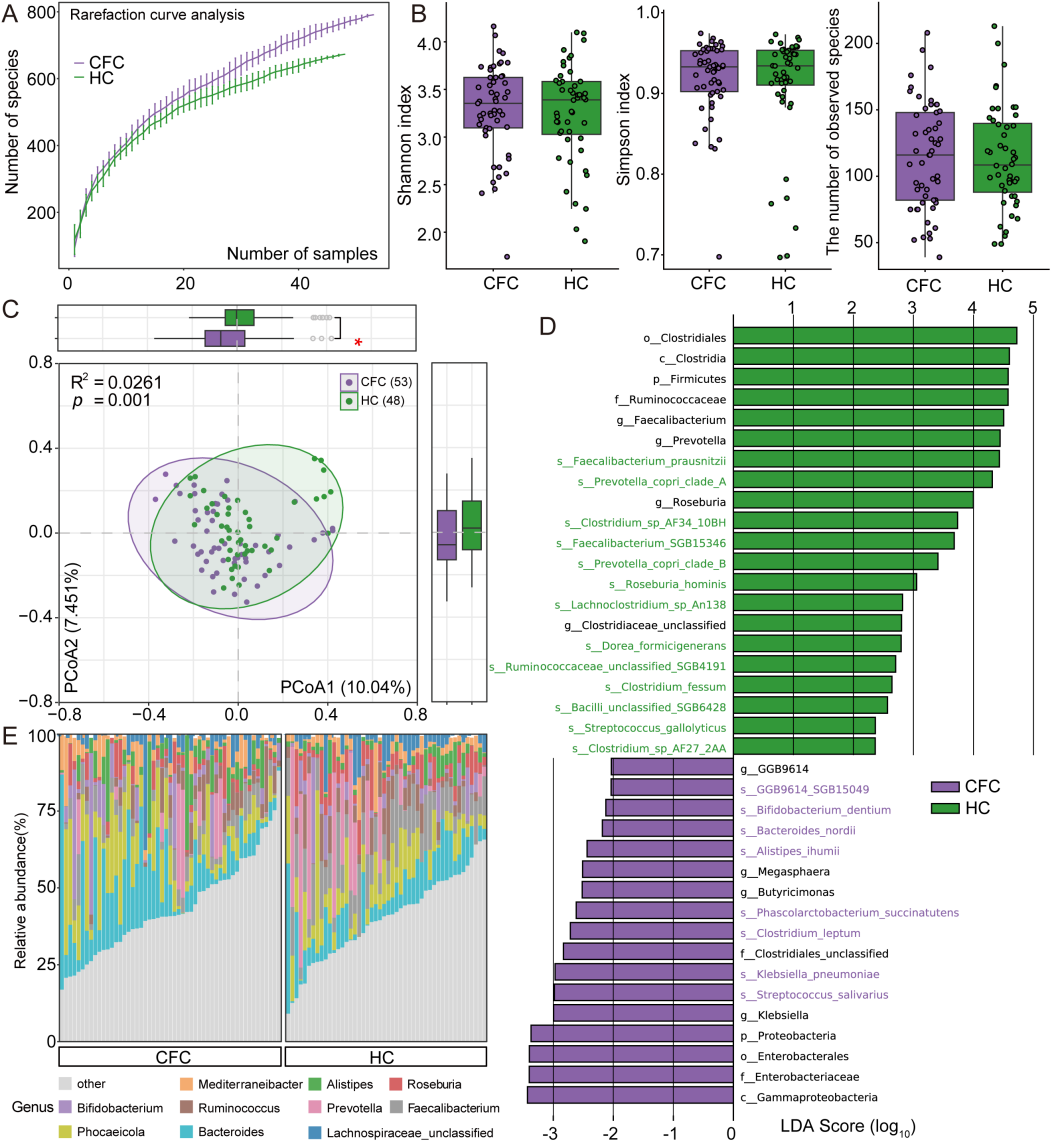
Gut bacteriome profiling in chronic functional constipation (CFC) patients and healthy controls (HC). **(A)** Rarefaction curves showing the number of observed bacterial species in each group. B) Boxplots of bacteriome alpha-diversity metrics, including the Shannon index, Simpson index, and observed species. **(C)** Principal coordinates analysis (PCoA) of Bray–Curtis dissimilarities based on species-level bacteriome profiles; each point represents one sample, with lines connecting samples within the same group. Circles indicate the group centroid and its surrounding dispersion, and the first two principal coordinates are shown. **(D)** LEfSe results identifying discriminative bacterial taxa between groups (thresholds: p < 0.05, LDA > 2.0); bar length indicates the LDA score (log10), and colors denote the group in which each taxon is relatively more abundant. **(E)** Genus-level taxonomic composition showing the top 10 most abundant genera across samples. Group differences were assessed using the Wilcoxon rank-sum test with Benjamini–Hochberg adjustment. *p < 0.05.

To investigate gut microbiome characteristics in 53 CFC patients and 48 HC, we performed shotgun metagenomic sequencing and obtained 205.8 Gbp of high-quality data (mean 2.0 ± 0.47 Gbp per sample). Reads were mapped to the UHGG database, yielding a taxonomic profile comprising 890 bacterial species across 17 phyla, 77 classes, 94 orders, 123 families, 464 genera, and 890 species. Rarefaction curves showed that species richness increased with sample size in both groups but did not reach saturation (**Figure 1A**), indicating that larger cohorts may be required to capture the full diversity. In contrast, α-diversity metrics (Shannon, Simpson, and richness) did not differ significantly between groups (Wilcoxon rank-sum test, *p* > 0.05; **Figure 1B**). We next assessed overall community structure using PCoA, which revealed a clear separation between groups (PERMANOVA, R² = 0.0621, *p* = 0.001; **Figure 1C**), indicating significant differences in global microbiome composition. LEfSe analysis (p < 0.05, LDA > 2.0) identified 38 differentially abundant taxa spanning 2 phyla, 2 classes, 2 orders, 3 families, 8 genera, and 21 species (**Figure 1D**, **Supplementary Table 2**). At the phylum level, Firmicutes were enriched in HC, whereas *Proteobacteria* were enriched in CFC. At the genus level, *Faecalibacterium*, *Prevotella*, *Roseburia*, and an *unclassified Clostridiaceae* genus were enriched in HC, while *GGB9614*, *Megasphaera*, *Butyricimonas*, and *Klebsiella* were enriched in CFC. At the species level, 13 species were enriched in HC (including *Faecalibacterium prausnitzii*, *Prevotella copri* clade_A, *Clostridium* sp. AF34_10BH, and *Faecalibacterium* SGB15346), whereas 8 species were enriched in CFC (including GGB9614_SGB15049, *Bifidobacterium dentium*, *Bacteroides nordii*, and *Alistipes ihumii*). Finally, among the top 10 most abundant genera, *Bacteroides* was the most abundant, followed by *Phocaeicola*, *Faecalibacterium*, and *Prevotella* (**Figure 1E**). Notably, *Faecalibacterium*, *Prevotella*, and *Roseburia* were significantly enriched in HC (**Supplementary Table 2**).

### Characteristics of the gut mycobiome in patients with CFC

**Figure 2:**
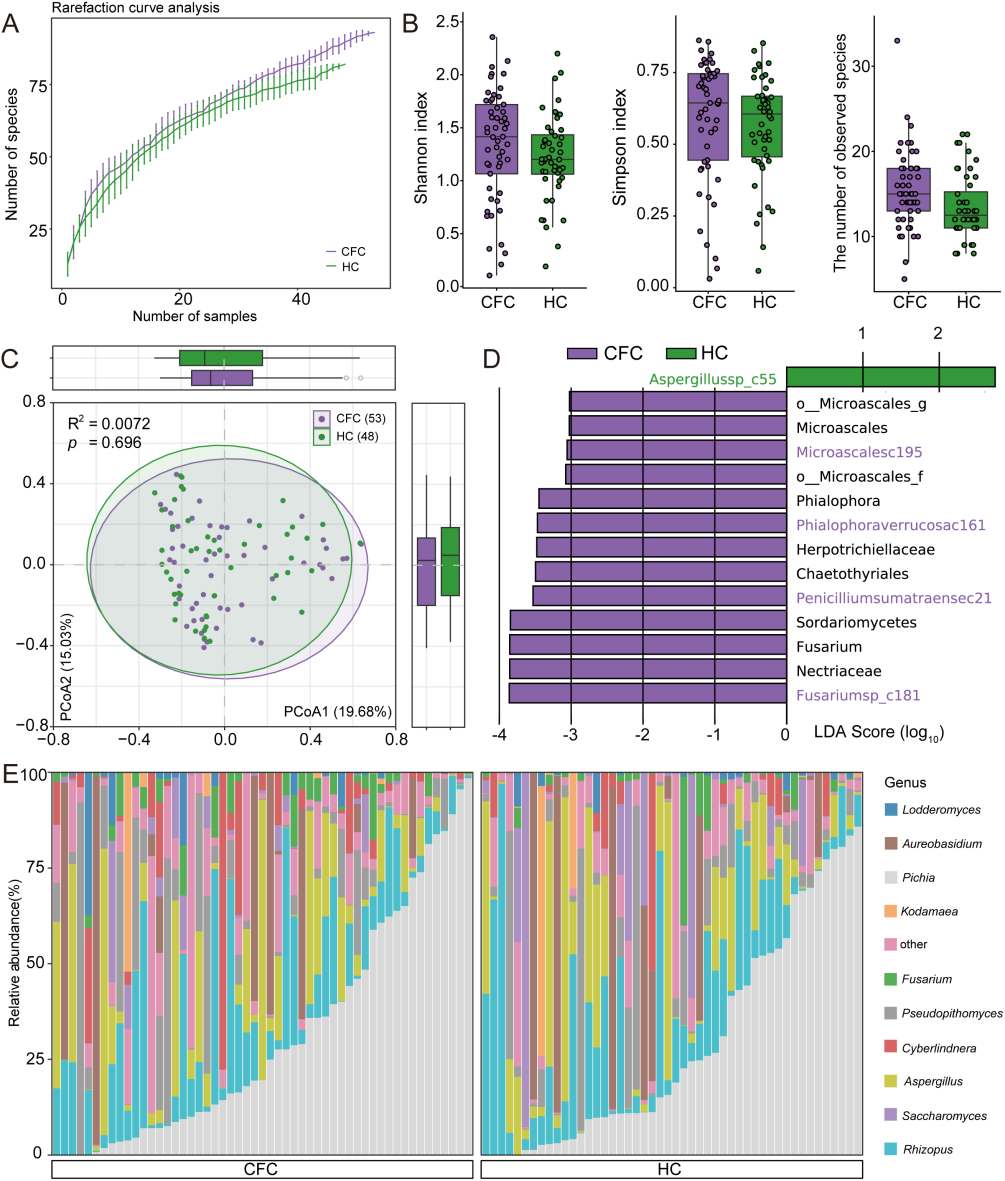
Gut mycobiome profiling in chronic functional constipation (CFC) patients and healthy controls (HC). **(A)** Rarefaction curves showing the number of observed fungal species in each group. **(B)** Boxplots of mycobiome alpha-diversity metrics, including the Shannon index, Simpson index, and observed species. **(C)** Principal coordinates analysis (PCoA) of Bray–Curtis dissimilarities based on species-level mycobiome profiles; each point represents one sample, with lines connecting samples within the same group. Circles indicate the group centroid and its surrounding dispersion, and the first two principal coordinates are shown. **(D)** LEfSe results identifying discriminative fungal taxa between groups (thresholds: p < 0.05, LDA > 2.0); bar length indicates the LDA score (log10), and colors denote the group in which each taxon is relatively more abundant. **(E)** Genus-level taxonomic composition showing the top 10 most abundant genera across samples. Group differences were assessed using the Wilcoxon rank-sum test with Benjamini–Hochberg adjustment.

To characterize the gut mycobiome in CFC, we profiled fungal communities in fecal metagenomes using a reference genome dataset covering 55 genera and 109 species, and compared the resulting profiles between CFC and HC. Rarefaction curves indicated that fungal species richness increased with sample size in both groups but did not reach saturation even at the maximum sampling depth (**Figure 2A**). Unlike the bacteriome, α-diversity differed between groups: both the Shannon index and richness were significantly different between CFC and HC (Wilcoxon rank-sum test, *p* < 0.05), whereas the Simpson index showed no significant difference (**Figure 2B**). In contrast, PCoA revealed no separation in overall mycobiome community structure between groups (PERMANOVA, R² = 0.00722, *p* = 0.696; **Figure 2C**). LEfSe analysis (*p* < 0.05, LDA > 2.0) identified 14 differentially abundant fungal taxa spanning 1 class, 2 orders, 3 families, 3 genera, and 5 species (**Figure 2D**; **Supplementary Table 3**). At the species level, only one species (*Aspergillus* sp. c55) was enriched in HC, whereas four species were enriched in CFC, including *Microascales c195, Phialophora verrucosa c161, Penicillium sumatraense c21*, and *Fusarium* sp. *c181*. At higher taxonomic ranks, a total of **nine taxa** were significantly enriched in the CFC group. Finally, among the top 10 most abundant fungal genera, *Pichia* was the most abundant, followed by *Rhizopus*, *Aspergillus*, and *Pseudopithomyces* (**Figure 2E**). Notably, *Fusarium* was significantly enriched in CFC.

### Characteristics of the gut virome in patients with CFC

**Figure 3:**
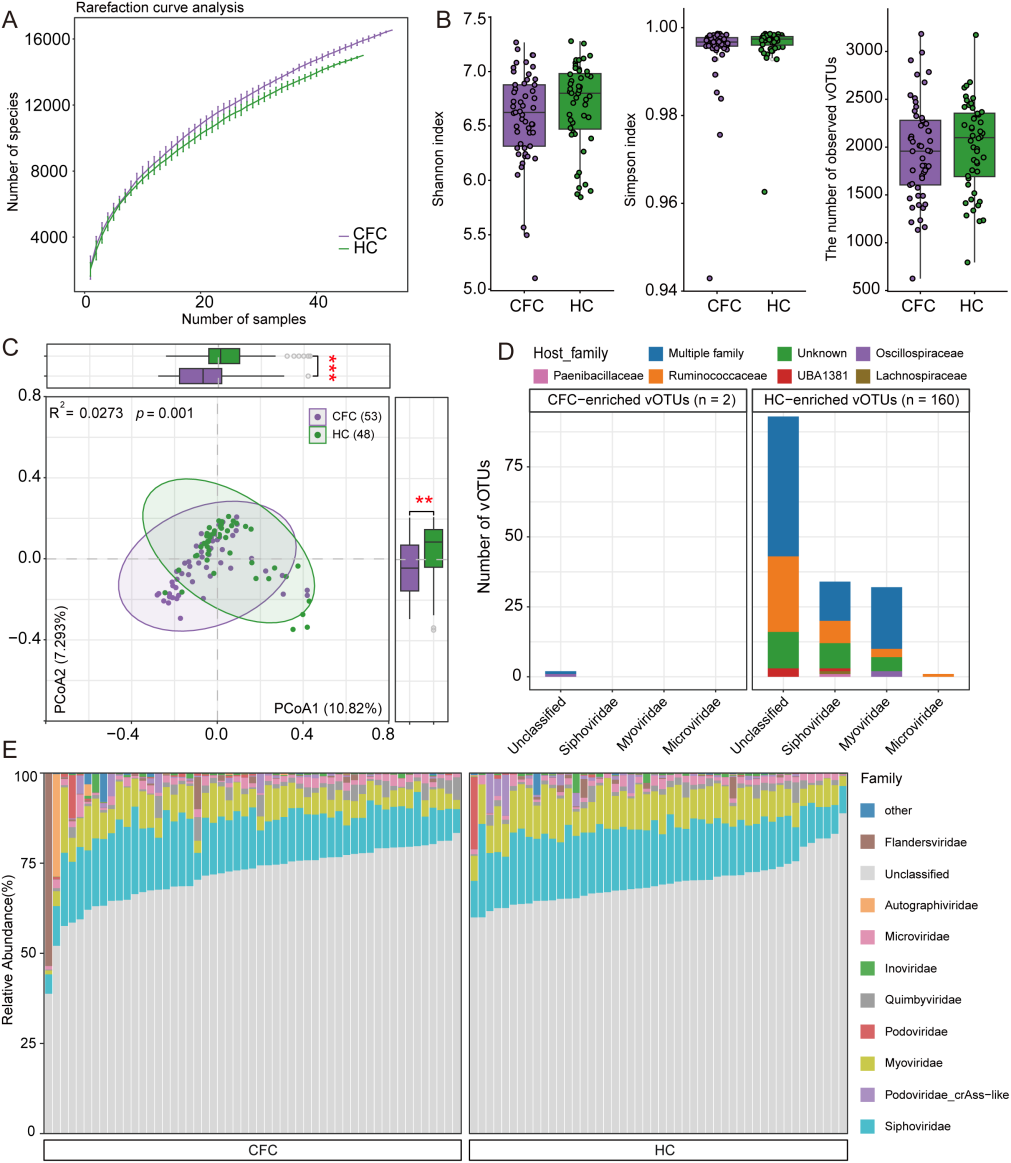
Gut virome profiling in chronic functional constipation (CFC) patients and healthy controls (HC). **(A)** Rarefaction curves showing the number of observed viral operational taxonomic units (vOTUs) in each group. **(B)** Boxplots of virome alpha-diversity metrics, including the Shannon index, Simpson index, and observed vOTUs. **(C)** Principal coordinates analysis (PCoA) of Bray–Curtis dissimilarities based on virome composition; each point represents one sample, with lines connecting samples within the same group. Circles indicate the group centroid and its surrounding dispersion, and the first two principal coordinates are shown. **(D)** Taxonomic annotation and predicted host assignment of group-differential vOTUs; vOTUs are summarized at the viral family level, and predicted prokaryotic hosts are shown at the genus level. **(E)** Family-level taxonomic composition showing the top 10 most abundant viral families across samples. Group differences were assessed using the Wilcoxon rank-sum test with Benjamini–Hochberg adjustment. ***p < 0.001.

To characterize the gut virome in CFC, we identified 20,701 vOTUs from metagenome-assembled contigs, ranging from 5,000 bp to 492,989 bp in length (mean 45,817 bp). Rarefaction analysis indicated higher vOTU richness in the CFC group than in HC at the same sampling depth (**Figure 3A**). However, α-diversity metrics (Shannon, Simpson, and richness) did not differ significantly between groups (Wilcoxon rank-sum test, *p* > 0.05; **Figure 3B**). In contrast, Bray–Curtis distance–based PCoA showed a clear separation of virome composition between CFC and HC (PERMANOVA, R² = 0.0273, *p* < 0.01), with significant differences along both PCoA1 and PCoA2 (**Figure 3C**). We examined virome differences at the genus level and identified 47 viral genera showing nominal group differences. However, because a substantial fraction of vOTUs lacked genus-level annotations, only Oengusvirus was significantly depleted in the CFC group (Wilcoxon rank-sum test, q < 0.05, |Fold change| > 1.2, minimum average relative abundance > 0.01; **Supplementary Table 4**). We therefore proceeded with a vOTU-level analysis for higher-resolution profiling. Differential abundance analysis identified 162 vOTUs that differed significantly between groups (Wilcoxon rank-sum test, *q* < 0.05, |Fold change| > 1.2, minimum average relative abundance > 0.01; **Figure 3D**, **Supplementary Table 5**). Of these, 160 vOTUs were enriched in HC, including 93 unclassified vOTUs, followed by taxa assigned to *Siphoviridae*, *Myoviridae*, and *Microviridae*. Predicted hosts were mainly bacteria spanning multiple families, including Ruminococcaceae, as well as unclassified taxa. In contrast, only two unclassified viral families were enriched in CFC, with predicted hosts primarily belonging to multiple families and Oscillospiraceae. Overall, taxonomic profiling showed that *Siphoviridae* was the most abundant viral family in both groups, followed by *Myoviridae* (**Figure 3E**).

### Functional alterations of the gut microbiome in CFC patients

**Figure 4:**
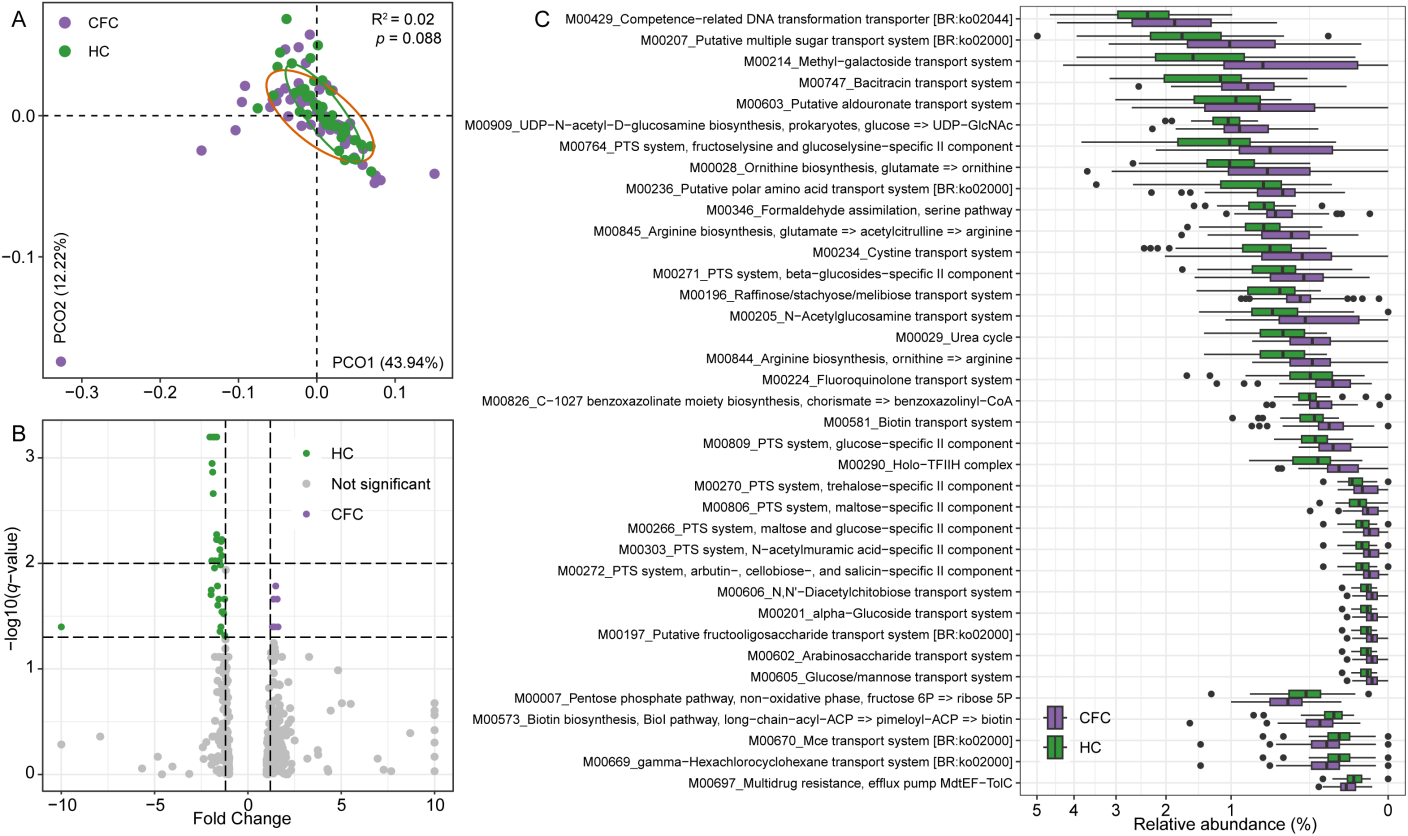
Functional profiling of the gut microbiome in patients with chronic functional constipation (CFC) and healthy controls (HC). **(A)** Principal coordinates analysis (PCoA) based on Bray–Curtis distances of KEGG functional module profiles. **(B)** Volcano plot showing differentially abundant KEGG functional modules between CFC and HC. **(C)** Relative abundances of representative KEGG functional modules that are significantly depleted or enriched in CFC patients. Statistical significance was assessed using the Wilcoxon rank-sum test with false discovery rate correction (*q* < 0.05, FC >1.2).

To explore functional differences in the gut microbiome between CFC patients and healthy controls, we annotated a total of 533 KEGG functional modules from shotgun metagenomic data and compared their abundance profiles between groups. PCoA based on Bray–Curtis distances showed substantial overlap between the CFC and control groups, with no statistically significant separation (R² = 0.02, *p* = 0.088), indicating that global functional profiles were largely similar between groups (**Figure 4A**). Differential abundance analysis identified 50 functional modules that differed significantly between groups (Wilcoxon rank-sum test, *q* < 0.05; **Figure 4B**; **Supplementary Table 6**). Among these, 42 modules were significantly depleted in CFC patients, whereas 8 modules were significantly enriched. Functional modules reduced in CFC were predominantly related to carbohydrate uptake and utilization, including multiple PTS transport systems (e.g., glucose-, maltose-, trehalose-, and beta-glucoside–specific systems), oligosaccharide transport systems (raffinose/stachyose/melibiose, fructooligosaccharides, arabinosaccharides), and amino sugar metabolism, such as *N*-acetylglucosamine and *N*-acetylmuramic acid transport. Several amino acid metabolisms–related pathways, including arginine and ornithine biosynthesis, as well as urea cycle–associated modules, were also significantly reduced in CFC patients (**Figure 4C**). In contrast, the modules enriched in CFC patients were mainly associated with stress response and metabolic reprogramming, including multidrug resistance efflux pumps (MdtEF–TolC), xenobiotic transport systems, pentose phosphate pathway (non-oxidative phase), and biotin biosynthesis via the BioI pathway. Collectively, these results suggest modest, feature-level differences in gut microbial functional capacity in CFC, including reduced carbohydrate transport and nutrient acquisition functions and relatively higher abundance of pathways related to microbial stress adaptation and alternative metabolic processes.

### Correlation analysis of multi-kingdom gut microbiota

**Figure 5:**
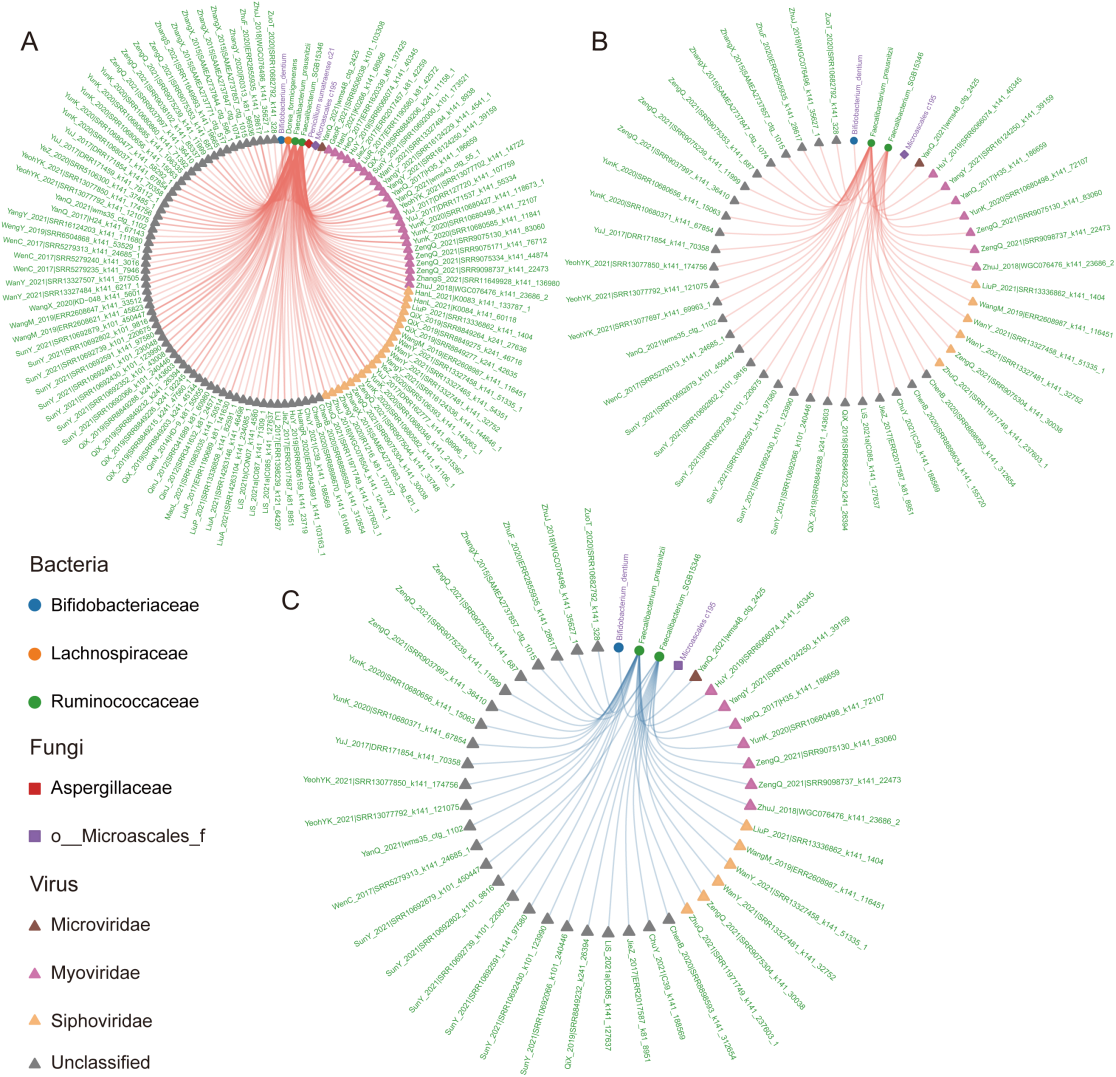
Correlation analysis among multi-kingdom microbial features. **(A)** Network showing correlations among multi-kingdom microbes in CFC patients. **(B)** Network showing correlations among multi-kingdom microbes in the HC group. **(C)** Correlations shared between the CFC and HC networks. In all three networks, different node shapes represent bacteria (circles), fungi (squares), and viruses (triangles). Node colors correspond to their family-level taxonomic classification. Microbial labels are colored based on their enrichment in CFC patients (purple) or HC (green).

To investigate cross-kingdom correlation patterns among gut bacteria, fungi, and viruses, we focused on 21 differentially abundant bacterial taxa, 5 differentially abundant fungal taxa, and 162 differentially abundant viral taxa (**Supplementary Table 7**). In the CFC group, we identified 179 significant correlations (Spearman |ρ| > 0.6), involving 119 taxa (4 bacterial, 2 fungal, and 113 viral taxa; **Supplementary Table 8**). Notably, all correlations were positive. Network analysis highlighted *Faecalibacterium*_*SGB15346* (degree = 83), *Faecalibacterium prausnitzii* (degree = 76), and *Dorea formicigenerans* (degree = 18) as highly connected hubs (**Supplementary Table 8**, **Figure 5A**). In contrast, the HC group exhibited substantially fewer cross-domain correlations, with only 44 significant relationships involving 47 taxa (3 bacterial, 1 fungal, and 43 viral taxa; **Supplementary Table 9**). Within the HC network, *Faecalibacterium prausnitzii* (degree = 29) and *Faecalibacterium*_*SGB15346* (degree = 14) showed the highest connectivity (**Supplementary Table 7**, **Figure 5B**). We further examined the 40 interactions shared between groups, which involved 111 taxa (3 bacterial, 1 fungal, and 39 viral taxa; **Supplementary Table 10**). Consistently, *Faecalibacterium prausnitzii* (degree = 25) and *Faecalibacterium*_*SGB15346* (degree = 14) remained the central nodes in the shared network (**Supplementary Table 7**, **Figure 5C**). Notably, these core taxa belong to the family Ruminococcaceae, consistently emerging as central nodes in the multi-kingdom co-occurrence networks, indicating robust cross-kingdom associations involving this lineage.

### Classification of CFC status based on gut microbiome

**Figure 6:**
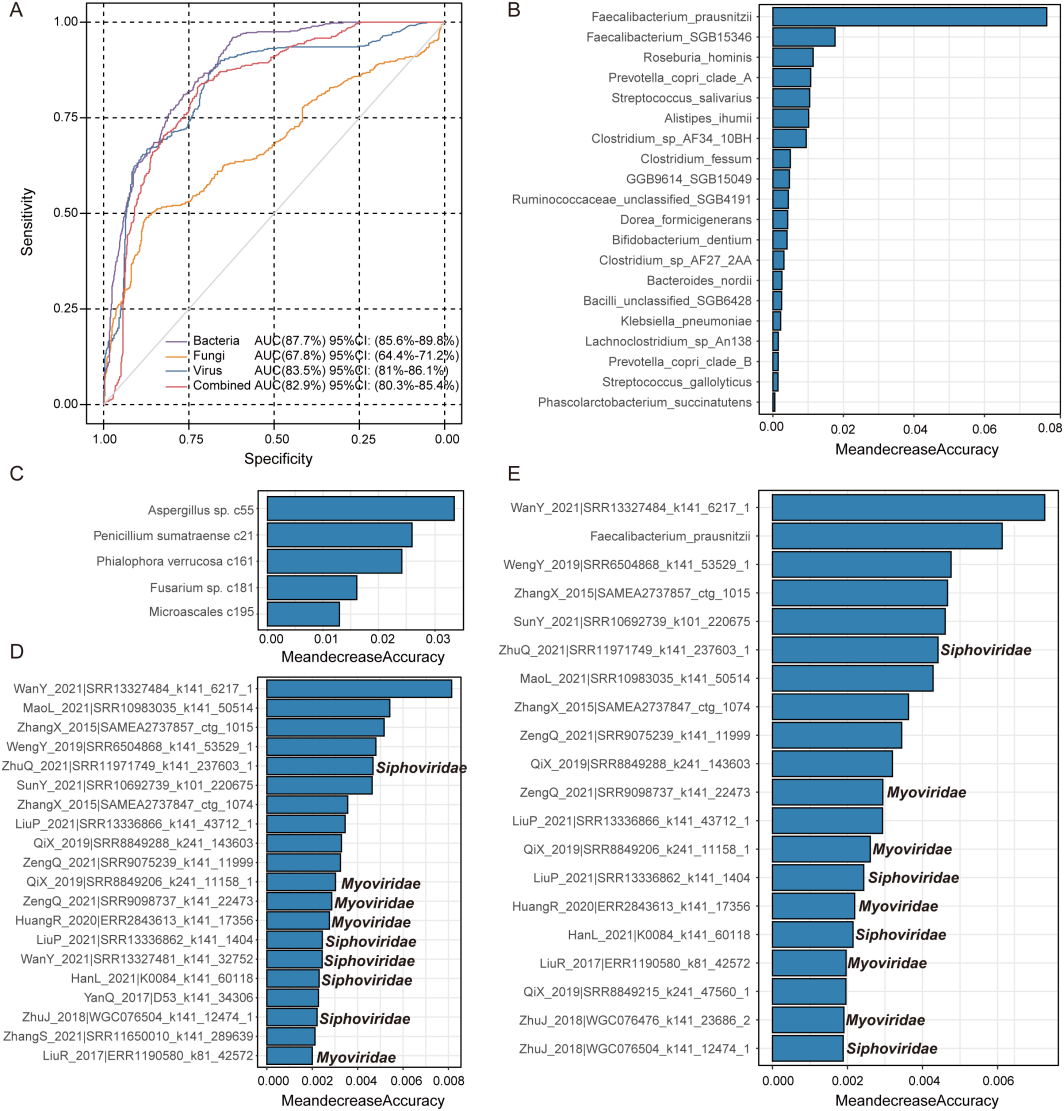
Classification of CFC patients and healthy controls (HC) based on gut multi-kingdom features. **(A)** ROC analysis evaluating the performance of bacterial, fungal, and viral features in classifying disease status. **(B–D)** The discriminative features in models based on bacteria **(B)**, fungi **(C)**, viruses **(D)**, and their combined multi-kingdom model **(E)**. Bar lengths represent the importance of each microbial feature. Black labels within the bar plot indicate the family-level taxonomic classification of the viruses.

We applied random forest classifiers to distinguish CFC patients from healthy controls and to evaluate the predictive value of gut microbial features across different kingdoms. Models trained on single-kingdom features showed distinct discriminatory performance. The bacterial-based model achieved the highest accuracy (AUC = 87.7%, 95% CI: 85.6%–89.8%), followed by the viral-based model (AUC = 83.5%, 95% CI: 81.0%–86.1%), whereas the fungal-based model showed more modest performance (AUC = 67.8%, 95% CI: 64.4%–71.2%) (**Figure 6A**). Notably, the combined multi-kingdom model did not improve predictive performance (AUC = 82.9%, 95% CI: 80.3%–85.4%) and was outperformed by the bacteria-only and virus-only models. Feature importance analysis revealed that several bacterial taxa were dominant predictors, including *Faecalibacterium prausnitzii*, *Faecalibacterium*_*SGB15346*, *Roseburia hominis*, and *Prevotella copri clade*_*A* (**Figure 6B**). Among fungal features, *Aspergillus* sp. *c55, Penicillium sumatraense c21, Phialophora verrucosa c161*, and *Fusarium* sp. *c181* contributed most strongly to model performance (**Figure 6C**). Viral predictors were primarily composed of an unclassified vOTU (*WanY_2021|SRR13327484_k141_6217_1*) and multiple members of the *Siphoviridae* and *Myoviridae* families (**Figure 6D**). In the combined model, *Faecalibacterium prausnitzii* remained the most influential bacterial feature, together with several high-importance viral vOTUs affiliated with *Siphoviridae* and *Myoviridae* (**Figure 6E**), indicating that viral signals complement the dominant bacterial predictors but do not surpass them in CFC classification.

## Discussion

In this study, we systematically profiled the gut bacteriome, mycobiome, and virome in patients with CFC compared with HC using whole-metagenome shotgun sequencing. We evaluated microbial diversity and community structure using α-diversity metrics and PCoA, identified differentially abundant taxa with LEfSe, and assessed cross-kingdom associations through correlation-based network analysis. We further explored the diagnostic potential of microbial signatures by building random forest classifiers based on bacterial, fungal, viral, and combined multi-kingdom features. Together, this integrative framework delineates multi-kingdom microbial alterations associated with CFC and highlights candidate features with potential diagnostic relevance.

In the bacteriome, α-diversity did not differ significantly between groups, whereas PCoA indicated a distinct community structure in CFC relative to HC. LEfSe identified 38 differentially abundant bacterial taxa, with Firmicutes (including *Faecalibacterium* and *Roseburia*) enriched in HC and Proteobacteria (including *Klebsiella*) enriched in CFC. Notably, *Faecalibacterium prausnitzii*, a butyrate-producing taxon with anti-inflammatory potential, was significantly reduced in CFC, consistent with its reported roles in supporting gut barrier integrity and intestinal motility (Miquel et al., 2013; Louis and Flint, 2017). Conversely, enrichment of potentially pathogenic taxa such as *Klebsiella* may contribute to dysbiosis and promote inflammatory perturbations that can impair intestinal function (Shin et al., 2015; Jiang et al., 2022). These findings indicate that CFC is associated with a shift from health-associated, fermentation-related bacteria toward taxa more often linked to dysbiotic states.

The analysis of the gut mycobiome in CFC patients revealed that, although the overall fungal community structure did not show significant differences between the CFC and HC groups, LEfSe analysis identified 14 differentially abundant fungal taxa. Among these, *Aspergillus* sp. c55 was enriched in the HC group, whereas potentially pathogenic fungi, including *Phialophora verrucosa* c161, *Penicillium sumatraense* c21, and *Fusarium* sp. c181, were significantly enriched in the CFC group. The reduced abundance of the opportunistic pathogen *Aspergillus* sp. *c55* in CFC patients may be attributed to suppression under disease conditions (Barac et al., 2024). The enrichment of fungi such as *Penicillium sumatraense* c21 and *Phialophora verrucosa* c161 in CFC patients could exacerbate gut dysbiosis and inflammatory responses by producing metabolites or interacting with bacterial communities (Richard and Sokol, 2019). Fungi of the *Fusarium* genus, known to be associated with increased intestinal permeability and inflammatory responses, may contribute to the pathogenesis of CFC by disrupting gut barrier function or inducing immune responses (Springler et al., 2016; Tian et al., 2024). These findings suggest that alterations in specific fungal taxa within the gut mycobiome of CFC patients may play a role in the disease’s pathogenesis by affecting gut permeability and inflammation.

In the virome, rarefaction analysis suggested higher vOTU richness in CFC, and PCoA revealed a clear separation in virome composition between groups, despite no significant differences in Shannon or Simpson indices. Given that gut viral communities are dominated by bacteriophages, these compositional changes likely reflect altered phage–host dynamics and their influence on bacterial community stability. Major phage families such as *Siphoviridae* and *Myoviridae* may shape bacterial abundance and ecological balance through predation and horizontal gene transfer (Nishijima et al., 2022). In addition, the enrichment of numerous unclassified viral taxa in HC highlights the substantial “dark matter” of the gut virome and underscores the need for improved viral reference databases to better resolve their functional and ecological roles (Shkoporov et al., 2019; Nishijima et al., 2022). Overall, our virome results support an underappreciated contribution of viral community remodeling to CFC-associated gut ecosystem alterations.

Although PCoA did not show a statistically significant separation of overall functional profiles between CFC patients and healthy controls, differential abundance analysis revealed clear shifts in specific KEGG modules. Most modules depleted in CFC were related to carbohydrate transport and utilization, including multiple phosphotransferase systems (PTS) and oligosaccharide transport pathways, suggesting reduced capacity for microbial substrate uptake and energy harvesting. In parallel, modules involved in amino sugar metabolism and nitrogen-related pathways, such as arginine and ornithine metabolism and the urea cycle, were also decreased, pointing to broader impairment in microbial metabolic activity. Conversely, modules enriched in CFC were mainly associated with stress adaptation and metabolic reprogramming, including multidrug resistance efflux systems, xenobiotic transport, the non-oxidative pentose phosphate pathway, and biotin biosynthesis. Together, these findings suggest modest, feature-level functional differences in the gut microbiome of CFC, including reduced nutrient acquisition functions and relatively higher stress-response pathways, which may be associated with an altered intestinal environment.

The correlation analysis revealed distinct multi-kingdom interaction patterns in CFC. In the CFC group, we identified 179 significant positive correlations, with viral taxa constituting the majority of nodes and edges in the network. This is consistent with prior evidence that gut viral communities, particularly bacteriophages, can exert strong ecological control over microbial ecosystems under disease conditions (Wylie et al., 2013; Liang and Bushman, 2021). Notably, *Faecalibacterium prausnitzii* and *Faecalibacterium*_SGB15346 emerged as central hubs with high connectivity, further supporting the pivotal role of *Faecalibacterium* in maintaining gut homeostasis and anti-inflammatory balance (Miquel et al., 2013). Cross-kingdom associations involving fungi were relatively limited in both groups; nevertheless, fungi may still influence bacterial and viral dynamics through metabolic interactions and niche competition (Iliev and Underhill, 2013). Moreover, the shared interaction network between CFC and HC retained a set of conserved correlations, suggesting that certain core microbial relationships persist across health and disease. Importantly, the dominant hubs in the shared network belonged to Ruminococcaceae, consistent with the recognized contribution of this family to fiber degradation and short-chain fatty acid production (Fang et al., 2020; Mirzaei et al., 2021; Facchin et al., 2024).

Random forest modelling further supported the diagnostic relevance of multi-kingdom microbial signatures. The bacteria-only model achieved the highest performance (AUC = 87.7%), indicating that bacterial taxa provide the strongest discriminatory signal for CFC classification. This is in line with previous studies linking CFC-related dysbiosis to alterations in health-associated taxa such as *Faecalibacterium prausnitzii* and *Roseburia hominis* (Miquel et al., 2013; Lopez-Siles et al., 2017). The virus-only model also showed robust predictive power (AUC = 83.5%), with important features largely derived from *Siphoviridae*- and *Myoviridae*-affiliated vOTUs, reinforcing the notion that bacteriophages contribute to, or reflect, ecosystem-level dysbiosis (Shkoporov and Hill, 2019; Liang and Bushman, 2021). By contrast, the fungi-only model showed more modest performance (AUC = 67.8%), suggesting that fungal profiles alone may be less informative for classification. Nonetheless, several fungal taxa, including *Aspergillus* sp. c55, *Penicillium sumatraense* c21, and *Phialophora verrucosa* c161, ranked highly in feature importance analyses, supporting a potential, albeit secondary, role for fungi in shaping gut ecosystem dynamics (Iliev and Underhill, 2013; Richard and Sokol, 2019).

Several limitations should be acknowledged. First, rarefaction curves for the bacteriome, mycobiome, and virome did not reach saturation, indicating that the current sequencing depth and/or sample size may not fully capture the complete diversity, particularly rare taxa. Larger cohorts and deeper sequencing will be required to refine multi-kingdom microbial profiles in CFC. Second, virome analyses relied heavily on unclassified viral families, reflecting incomplete viral reference databases. In addition, only two unclassified viral families were enriched in CFC, which constrains biological interpretation of disease-associated viral signatures. Continued expansion of viral genome resources and improved host–virus annotation strategies will be essential to better resolve virome alterations and their functional implications in CFC.

## Conclusion

In summary, this shotgun metagenomic study provides an integrated multi-kingdom view of the gut microbiome in CFC. CFC was associated with pronounced community-structure shifts in both the bacteriome and virome, accompanied by specific taxonomic changes, including depletion of health-associated Firmicutes taxa (e.g., *Faecalibacterium* and *Roseburia*) and enrichment of Proteobacteria (e.g., *Klebsiella*). The mycobiome showed selective alterations in differential taxa despite broadly similar overall community structure. Although global functional profiles largely overlapped between groups, targeted KEGG modules differed, featuring widespread depletion of carbohydrate transport/utilization pathways and enrichment of stress-adaptation and metabolic reprogramming functions in CFC. Cross-kingdom network analysis further revealed substantially denser positive associations in CFC, with viral taxa dominating network connectivity and *Faecalibacterium prausnitzii* and *Faecalibacterium*_SGB15346 emerging as central hubs across networks, suggesting conserved yet rewired multi-domain interactions centered on Ruminococcaceae. Finally, random forest models demonstrated that bacterial and viral signatures provided strong discriminatory power for CFC classification, whereas fungal features were less informative and multi-kingdom integration did not improve performance. Together, these findings highlight coordinated multi-kingdom dysbiosis in CFC, with modest, feature-level functional alterations rather than a marked global functional shift, supporting the potential of microbiome-based biomarkers, while underscoring the need for larger cohorts and improved virome annotation to clarify ecological mechanisms and clinical relevance.

## Data Availability

The original contributions presented in the study are publicly available. These data can be found in the European Nucleotide Archive (ENA) under accession number PRJEB108281: https://www.ebi.ac.uk/ena/browser/view/PRJEB108281.

## References

[B1] Almeida A. Nayfach S. Boland M. Strozzi F. Beracochea M. Shi Z. J. . (2021). A unified catalog of 204,938 reference genomes from the human gut microbiome. Nat. Biotechnol. 39, 105–114. doi: 10.1038/s41587-020-0603-3, PMID: 32690973 PMC7801254

[B2] Barac A. Vujovic A. Peric J. Tulic I. Stojanovic M. Stjepanovic M. (2024). Rethinking aspergillosis in the era of microbiota and mycobiota. Mycopathologia 189, 49. doi: 10.1007/s11046-024-00853-2, PMID: 38864956

[B3] Belkaid Y. Hand T. W. (2014). Role of the microbiota in immunity and inflammation. Cell 157, 121–141. doi: 10.1016/j.cell.2014.03.011, PMID: 24679531 PMC4056765

[B4] Benninga M. Candy D. C. Catto-Smith A. G. Clayden G. Loening-Baucke V. Di Lorenzo C. . (2005). The Paris consensus on childhood constipation terminology (PACCT) group. J Pediatr Gastroenterol Nutr. 40, 273–5. 15735478 10.1097/01.mpg.0000158071.24327.88

[B5] Blanco-Míguez A. Beghini F. Cumbo F. McIver L. J. Thompson K. N. Zolfo M. . (2023). Extending and improving metagenomic taxonomic profiling with uncharacterized species using MetaPhlAn 4. Nat. Biotechnol. 41, 1633–1644. doi: 10.1038/s41587-023-01688-w, PMID: 36823356 PMC10635831

[B6] Camilleri M. Ford A. C. Mawe G. M. Dinning P. G. Rao S. S. Chey W. D. . (2017). Chronic constipation. Nat. Rev. Dis. Primers 3, 17095. doi: 10.1038/nrdp.2017.95, PMID: 29239347

[B7] Chen S. Zhou Y. Chen Y. Gu J. (2018). fastp: an ultra-fast all-in-one FASTQ preprocessor. Bioinformatics 34, i884–i890. doi: 10.1093/bioinformatics/bty560, PMID: 30423086 PMC6129281

[B8] Di Tommaso N. Gasbarrini A. Ponziani F. R. (2021). Intestinal barrier in human health and disease. Int. J. Environ. Res. Public Health 18, 12836. doi: 10.3390/ijerph182312836, PMID: 34886561 PMC8657205

[B9] Facchin S. Bertin L. Bonazzi E. Lorenzon G. De Barba C. Barberio B. . (2024). Short-chain fatty acids and human health: from metabolic pathways to current therapeutic implications. Life 14, 559. doi: 10.3390/life14050559, PMID: 38792581 PMC11122327

[B10] Fang S. Chen X. Ye X. Zhou L. Xue S. Gan Q. (2020). Effects of gut microbiome and short-chain fatty acids (SCFAs) on finishing weight of meat rabbits. Front. Microbiol. 11, 1835. doi: 10.3389/fmicb.2020.01835, PMID: 32849435 PMC7431612

[B11] Flint H. J. Scott K. P. Duncan S. H. Louis P. Forano E. (2012). Microbial degradation of complex carbohydrates in the gut. Gut Microbes 3, 289–306. doi: 10.4161/gmic.19897, PMID: 22572875 PMC3463488

[B12] Gregory A. C. Zayed A. A. Conceição-Neto N. Temperton B. Bolduc B. Alberti A. . (2019). Marine DNA viral macro-and microdiversity from pole to pole. Cell 177, 1109–1123. doi: 10.1016/j.cell.2019.03.040, PMID: 31031001 PMC6525058

[B13] Handelsman J. (2004). Metagenomics: application of genomics to uncultured microorganisms. Microbiol. Mol. Biol. Rev. 68, 669–685. doi: 10.1128/MMBR.68.4.669-685.2004, PMID: 15590779 PMC539003

[B14] Huse S. M. Ye Y. Zhou Y. Fodor A. A. (2012). A core human microbiome as viewed through 16S rRNA sequence clusters. PloS One 7, e34242. doi: 10.1371/journal.pone.0034242, PMID: 22719824 PMC3374614

[B15] Iliev I. D. Underhill D. M. (2013). Striking a balance: fungal commensalism versus pathogenesis. Curr. Opin. Microbiol. 16, 366–373. doi: 10.1016/j.mib.2013.05.004, PMID: 23756050 PMC3742553

[B16] Jiang Q. Xu Q. Kenéz Á. Chen S. Yang G. (2022). Klebsiella pneumoniae infection is associated with alterations in the gut microbiome and lung metabolome. Microbiological Res. 263, 127139. doi: 10.1016/j.micres.2022.127139, PMID: 35905579

[B17] Kanehisa M. Goto S. Sato Y. Kawashima M. Furumichi M. Tanabe M. (2014). Data, information, knowledge and principle: back to metabolism in KEGG. Nucleic Acids Res. 42, D199–D205. doi: 10.1093/nar/gkt1076, PMID: 24214961 PMC3965122

[B18] Langmead B. Salzberg S. L. (2012). Fast gapped-read alignment with Bowtie 2. Nat. Methods 9, 357–359. doi: 10.1038/nmeth.1923, PMID: 22388286 PMC3322381

[B19] Lembo A. Camilleri M. (2003). Chronic constipation. New Engl. J. Med. 349, 1360–1368. doi: 10.1056/NEJMra020995, PMID: 14523145

[B20] Liang G. Bushman F. D. (2021). The human virome: assembly, composition and host interactions. Nat. Rev. Microbiol. 19, 514–527. doi: 10.1038/s41579-021-00536-5, PMID: 33785903 PMC8008777

[B21] Liaw A. Wiener M. (2002). Classification and regression by randomForest. R News 2, 18–22. doi: 10.1023/A:1010933404324, PMID: 40177105

[B22] Lopez-Siles M. Duncan S. H. Garcia-Gil L. J. Martinez-Medina M. (2017). Faecalibacterium prausnitzii: from microbiology to diagnostics and prognostics. ISME J. 11, 841–852. doi: 10.1038/ismej.2016.176, PMID: 28045459 PMC5364359

[B23] Louis P. Flint H. J. (2017). Formation of propionate and butyrate by the human colonic microbiota. Environ. Microbiol. 19, 29–41. doi: 10.1111/1462-2920.13589, PMID: 27928878

[B24] Mancabelli L. Milani C. Lugli G. A. Turroni F. Mangifesta M. Viappiani A. . (2017). Unveiling the gut microbiota composition and functionality associated with constipation through metagenomic analyses. Sci. Rep. 7, 9879. doi: 10.1038/s41598-017-10663-w, PMID: 28852182 PMC5575163

[B25] Miquel S. Martín R. Rossi O. Bermúdez-Humarán L. G. Chatel J. M. Sokol H. . (2013). Faecalibacterium prausnitzii and human intestinal health. Curr. Opin. Microbiol. 16, 255–261. doi: 10.1016/j.mib.2013.06.003, PMID: 23831042

[B26] Mirzaei R. Bouzari B. Hosseini-Fard S. R. Mazaheri M. Ahmadyousefi Y. Abdi M. . (2021). Role of microbiota-derived short-chain fatty acids in nervous system disorders. Biomedicine Pharmacotherapy 139, 111661. doi: 10.1016/j.biopha.2021.111661, PMID: 34243604

[B27] Nagalingam N. A. Lynch S. V. (2012). Role of the microbiota in inflammatory bowel diseases. Inflammatory bowel Dis. 18, 968–984. doi: 10.1002/ibd.21866, PMID: 21936031

[B28] Nishijima S. Nagata N. Kiguchi Y. Kojima Y. Miyoshi-Akiyama T. Kimura M. . (2022). Extensive gut virome variation and its associations with host and environmental factors in a population-level cohort. Nat. Commun. 13, 5252. doi: 10.1038/s41467-022-32832-w, PMID: 36068216 PMC9448778

[B29] Ohkusa T. Koido S. Nishikawa Y. Sato N. (2019). Gut microbiota and chronic constipation: a review and update. Front. Med. 6, 19. doi: 10.3389/fmed.2019.00019, PMID: 30809523 PMC6379309

[B30] Öhman L. Simrén M. (2013). Intestinal microbiota and its role in irritable bowel syndrome (IBS). Curr. Gastroenterol. Rep. 15, 323. doi: 10.1007/s11894-013-0323-7, PMID: 23580243

[B31] Oksanen J. Kindt R. Legendre P. (2007). The vegan package. Community Ecol. Package 10, 719.

[B32] Pan R. Wang L. Xu X. Chen Y. Wang H. Wang G. . (2022). Crosstalk between the gut microbiome and colonic motility in chronic constipation: potential mechanisms and microbiota modulation. Nutrients 14, 3704. doi: 10.3390/nu14183704, PMID: 36145079 PMC9505360

[B33] Pinto Sanchez M. I. Bercik P. (2011). Epidemiology and burden of chronic constipation. Can. J. Gastroenterol. Hepatol. 25, 11B–15B. doi: 10.1155/2011/974573, PMID: 22114752 PMC3206560

[B34] Qin J. Li R. Raes J. Arumugam M. Burgdorf K. S. Manichanh C. . (2010). A human gut microbial gene catalogue established by metagenomic sequencing. Nature 464, 59–65. doi: 10.1038/nature08821, PMID: 20203603 PMC3779803

[B35] Quast C. Pruesse E. Yilmaz P. Gerken J. Schweer T. Yarza P. . (2012). The SILVA ribosomal RNA gene database project: improved data processing and web-based tools. Nucleic Acids Res. 41, D590–D596. doi: 10.1093/nar/gks1219, PMID: 23193283 PMC3531112

[B36] Richard M. L. Sokol H. (2019). The gut mycobiota: insights into analysis, environmental interactions and role in gastrointestinal diseases. Nat. Rev. Gastroenterol. Hepatol. 16, 331–345. doi: 10.1038/s41575-019-0121-2, PMID: 30824884

[B37] Robin X. Turck N. Hainard A. Tiberti N. Lisacek F. Sanchez J. C. . (2011). pROC: an open-source package for R and S+ to analyze and compare ROC curves. BMC Bioinf. 12, 77. doi: 10.1186/1471-2105-12-77, PMID: 21414208 PMC3068975

[B38] Sbahi H. Cash B. D. (2015). Chronic constipation: a review of current literature. Curr. Gastroenterol. Rep. 17, 47. doi: 10.1007/s11894-015-0471-z, PMID: 26449614

[B39] Segata N. Izard J. Waldron L. Gevers D. Miropolsky L. Garrett W. S. . (2011). Metagenomic biomarker discovery and explanation. Genome Biol. 12, R60. doi: 10.1186/gb-2011-12-6-r60, PMID: 21702898 PMC3218848

[B40] Shin N. R. Whon T. W. Bae J. W. (2015). Proteobacteria: microbial signature of dysbiosis in gut microbiota. Trends Biotechnol. 33, 496–503. doi: 10.1016/j.tibtech.2015.06.011, PMID: 26210164

[B41] Shkoporov A. N. Clooney A G. Sutton T. D. S. Ryan F J. Daly K. M. Nolan J. A. . (2019). The human gut virome is highly diverse, stable, and individual specific. Cell Host Microbe 26, 527–541. doi: 10.1016/j.chom.2019.09.009, PMID: 31600503

[B42] Shkoporov A. N. Hill C. (2019). Bacteriophages of the human gut: the “known unknown” of the microbiome. Cell Host Microbe 25, 195–209. doi: 10.1016/j.chom.2019.01.017, PMID: 30763534

[B43] Springler A. Vrubel G. J. Mayer E. Schatzmayr G. Novak B. (2016). Effect of Fusarium-derived metabolites on the barrier integrity of differentiated intestinal porcine epithelial cells (IPEC-J2). Toxins 8, 345. doi: 10.3390/toxins8110345, PMID: 27869761 PMC5127141

[B44] Su G. Morris J. H. Demchak B. Bader G. D. (2014). Biological network exploration with Cytoscape 3. Curr. Protoc. Bioinf. 47, 8.13.1–24. doi: 10.1002/0471250953.bi0813s47, PMID: 25199793 PMC4174321

[B45] Suares N. C. Ford A. C. (2011). Systematic review: the effects of fibre in the management of chronic idiopathic constipation. Alimentary Pharmacol. Ther. 33, 895–901. doi: 10.1111/j.1365-2036.2011.04602.x, PMID: 21332763

[B46] Thomas T. Gilbert J. Meyer F. (2012). Metagenomics-a guide from sampling to data analysis. Microbial Inf. experimentation 2, 3. doi: 10.1186/2042-5783-2-3, PMID: 22587947 PMC3351745

[B47] Tian X. Li S. Wang C. Zhang Y. Feng X. Yan Q. . (2024). Gut virome-wide association analysis identifies cross-population viral signatures for inflammatory bowel disease. Microbiome 12, 130. doi: 10.1186/s40168-024-01832-x, PMID: 39026313 PMC11256409

[B48] Turnbaugh P. J. Ley R. E. Hamady M. Fraser-Liggett C. M. Knight R. Gordon J. I. (2007). The human microbiome project. Nature 449, 804–810. doi: 10.1038/nature06244, PMID: 17943116 PMC3709439

[B49] Wickham H. Chang W. Wickham M. H. (2016). Package ‘ggplot2’. Create elegant data visualisations using the grammar of graphics. Book of Abstracts 2, 1–189.

[B50] Wylie K. M. Weinstock G. M. Storch G. A. (2013). Virome genomics: a tool for defining the human virome. Curr. Opin. Microbiol. 16, 479–484. doi: 10.1016/j.mib.2013.04.006, PMID: 23706900 PMC3755052

[B51] Yan Q. Huang L. Li S. Zhang Y. Guo R. Zhang P. . (2025). The Chinese gut virus catalogue reveals gut virome diversity and disease-related viral signatures. Genome Med. 17, 30. doi: 10.1186/s13073-025-01460-6, PMID: 40140988 PMC11938785

[B52] Yan Q. Li S. Yan Q. Huo X. Wang C. Wang X. . (2024). A genomic compendium of cultivated human gut fungi characterizes the gut mycobiome and its relevance to common diseases. Cell 187, 2969–2989. doi: 10.1016/j.cell.2024.04.043, PMID: 38776919

[B53] Yang L. Wang Y. Zhang Y. Li W. Jiang S. Qian D. . (2022). Gut microbiota: a new avenue to reveal pathological mechanisms of constipation. Appl. Microbiol. Biotechnol. 106, 6899–6913. doi: 10.1007/s00253-022-12197-2, PMID: 36190540

[B54] Zhao Y. Yu Y. B. (2016). Intestinal microbiota and chronic constipation. Springerplus 5, 1130. doi: 10.1186/s40064-016-2821-1, PMID: 27478747 PMC4951383

