# Supplementary figures and images for: Multi-kingdom metagenomic characterization of the gut bacteriome, mycobiome, and virome in chronic functional constipation
Source: Front Cell Infect Microbiol. 2026 Mar 23;16:1744020. doi: 10.3389/fcimb.2026.1744020 (PMC13050957; doi:10.3389/fcimb.2026.1744020)

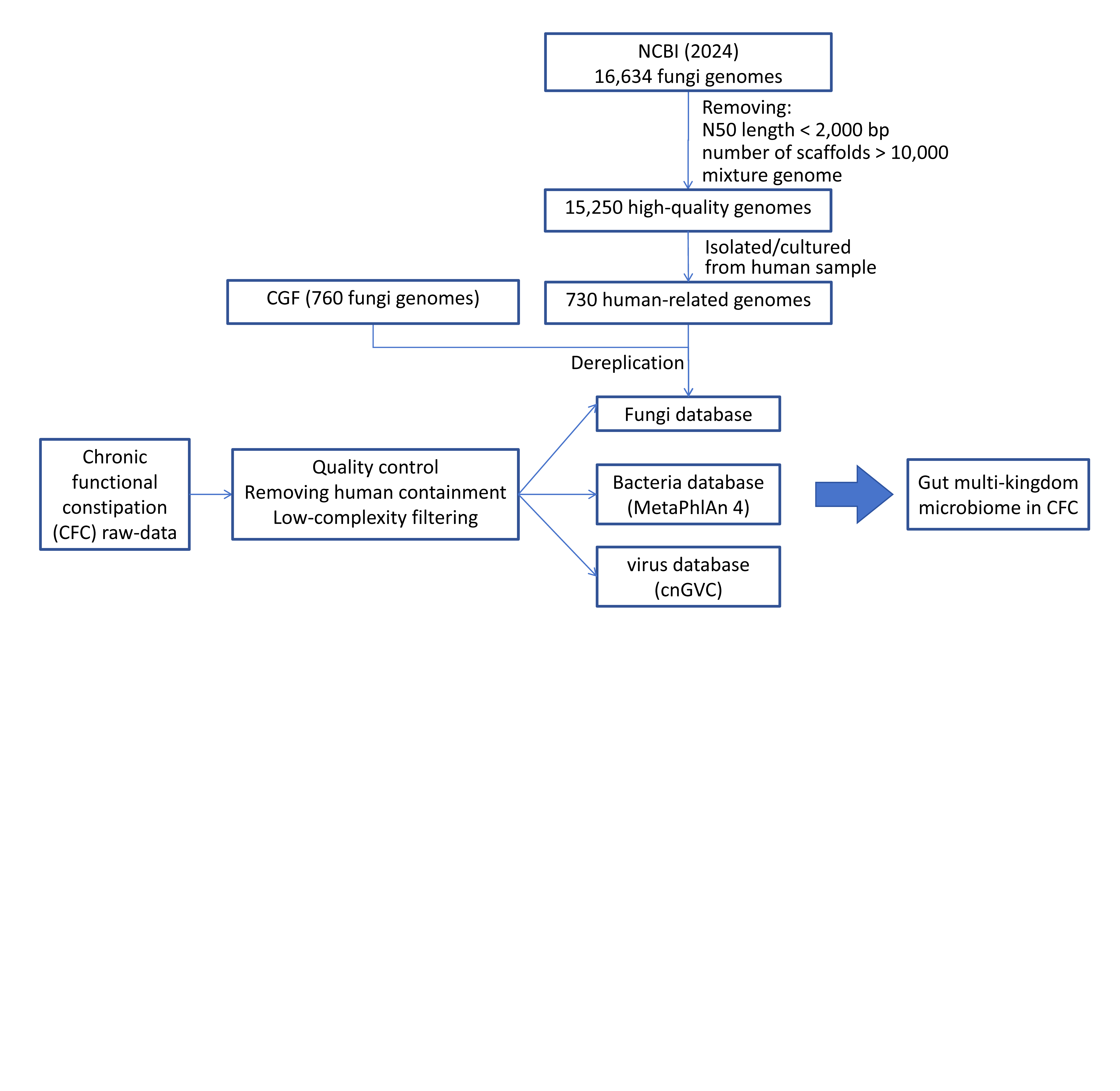

Supplement: Supplementary file 1 [file Image1.tif]

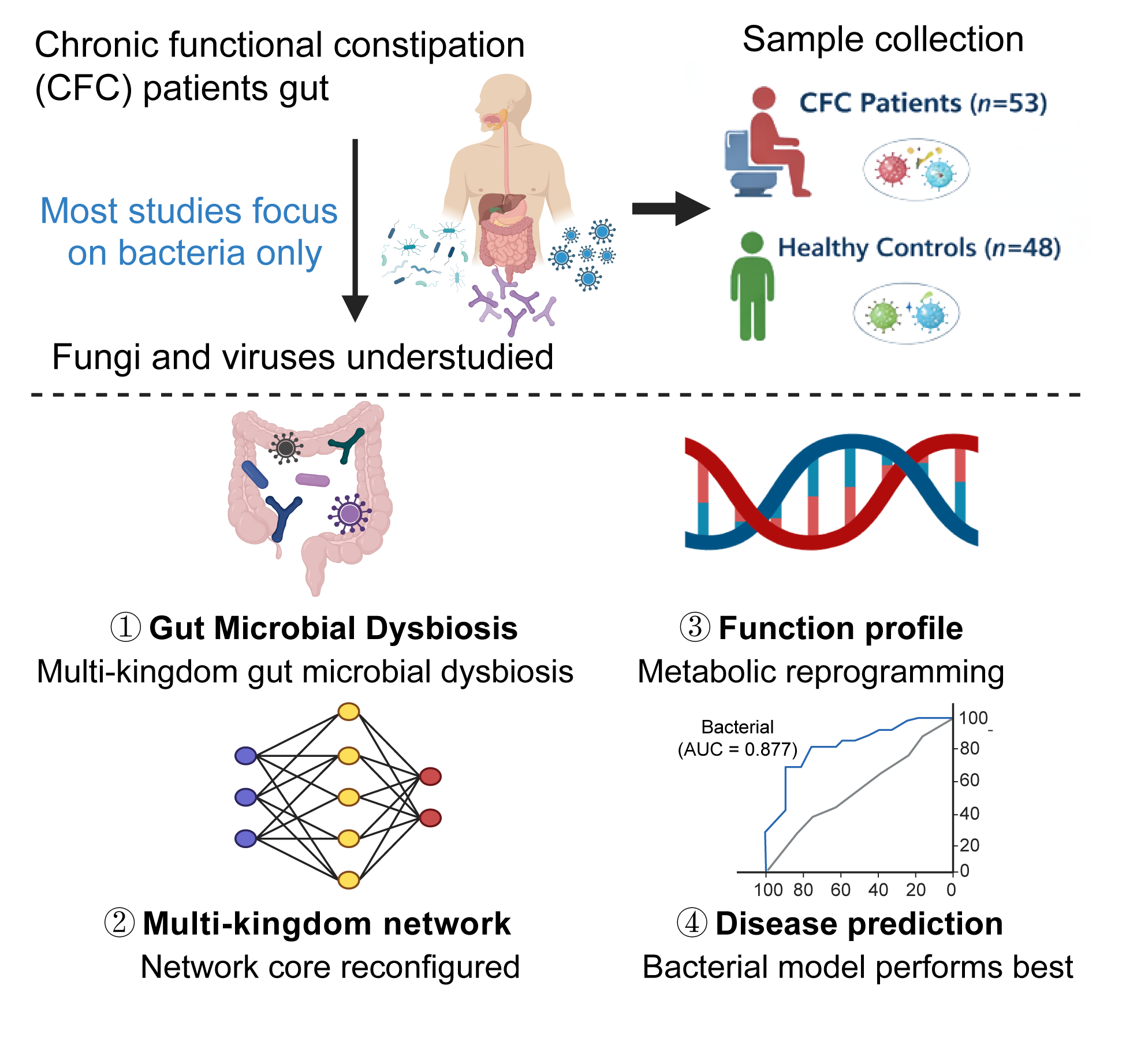

Supplement: Supplementary file 2 [file Image2.tif]
